# Mathematical analysis of the effect of process conditions on the porous structure development of activated carbons derived from Pine cones

**DOI:** 10.1038/s41598-022-19383-2

**Published:** 2022-09-12

**Authors:** Mirosław Kwiatkowski, Edward Gómez-Delgado, Gisel Vanesa Nunell, Pablo Ricardo Bonelli, Ana Lea Cukierman

**Affiliations:** 1 Faculty of Energy and Fuels, AGH University of Science and Technology, 30 Mickiewicza Avenue, 30-059 Krakow, Poland; 2 Depto. de Industrias, Facultad de Ciencias Exactas y Naturales, Instituto de Tecnología de Alimentos y Procesos Químicos ITAPROQ, CONICET, Universidad de Buenos Aires, Int. Güiraldes 2620, Ciudad Universitaria, (C1428BGA), Buenos Aires, Argentina; 3 Depto. de Tecnología Farmacéutica, Facultad de Farmacia y Bioquímica, Cátedra de Tecnología Farmacéutica II, Universidad de Buenos Aires, Junín 956, (C1113AAD), Buenos Aires, Argentina

**Keywords:** Chemistry, Materials science, Mathematics and computing, Physics

## Abstract

This paper presents the results of a study on the influence of the degree of impregnation and activation temperature on the formation of the porous structure of activated carbons (ACs) obtained from Pine cones by the chemical activation process using potassium hydroxide as an activator. The advanced new numerical clustering based adsorption analysis (LBET) method, together with the implemented unique numerical procedure for the fast multivariant identification were applied to nitrogen and carbon dioxide adsorption isotherms determined for porous structure characterization of the ACs. Moreover, the Quenched Solid Density Functional Theory (QSDFT) method was chosen to determine pore size distributions. The results showed a significant influence of the primary structure of Pine cones on the formation of the porous structure of the developed ACs. Among others, it was evidenced by a very high degree of surface heterogeneity of all the obtained ACs, irrespective of the degree of impregnation with potassium hydroxide and the activation temperature. Moreover, the analysis of carbon dioxide adsorption isotherms showed, that the porous structure of the studied ACs samples contains micropores accessible only to carbon dioxide molecules. The results also showed a significant advantage of the LBET method over those conventionally used for porous structure analysis based on Brunauer–Emmett–Teller (BET) and Dubinin–Raduskevich (DR) equations, because it takes into account surface heterogeneities. The novel analyses methods were more fully validated as a reliable characterization tool, by extending their application to the isotherms for ACs developed from the same precursor by phosphoric acid activation, and for samples arising from these ACs, further subjected to additional post-treatments. The effect of the raw material used as precursor was moreover analysed by comparison with previous reported results for other ACs. The complementarity of the results obtained with the LBET and QSDFT methods is also noteworthy, resulting in a more complete and reliable picture of the analyzed porous structures.

## Introduction

One of the greatest threats to the natural environment is the emission of harmful substances to the atmosphere and ground waters. Hence, a great number of measures have been taken to reduce degradation of the natural environment. Among them, adsorption using microporous activated carbons (ACs) has attracted increasing attention^1–16^.

The increase in the use of activated carbons observed in recent years is caused by their unique physicochemical properties, including a developed specific surface area, high adsorption capacity, mechanical strength and chemical resistance. An advantageous feature of activated carbons in comparison with other adsorbents is their ease of regeneration, relatively low cost production and large resources of raw materials^17^.

The precursors for the production of activated carbons can be any carbonaceous feedstock. However, they should be characterised by high content of elemental carbon, low content of volatiles, and inorganic substances, as well as high mechanical and thermal resistance, together with easy availability and low price. Currently, there is a very high interest in obtaining activated carbons from biomass wastes from forestry, wood and food industries. Use of waste biomass for the production of carbonaceous adsorbents, not only allows to obtain high quality adsorbents, but also contributes to waste management that often poses a significant threat to the natural environment^17–26^.

Activated carbons are basically obtained by the so-called physical or chemical activation process^27^. Physical activation is commonly used on an industrial scale for the production of activated carbons, due to relatively low production costs and no need for chemical substances^27,28^. It involves carbonization of the precursor in an inert atmosphere followed by the subsequent partial gasification of the carbonized precursor with a mild oxidizing agent, such as steam or CO_2_, at high temperatures (1073–1273 K) under strictly controlled conditions^27,28^.

Chemical activation consists of impregnation of the raw material selected as a precursor with a suitable chemical agent^29^. The mixture prepared in this way is then thermally treated at relatively moderate temperatures. In this process, the resulting solid products formed by mixtures of the remaining carbon and other side products require to be washed in order to eliminate the latter and to obtain a completely developed free pore structure^29,30^. An important advantage of chemical activation is that the process normally takes place at a lower temperature and shorter time than those used in physical activation. Moreover, yields in chemical activation are usually higher than in physical activation because the chemical agents used possess dehydrogenation properties that inhibit formation of tar and reduce the production of other volatile products^12^. In addition, activated carbons obtained by chemical activation have a larger surface area and well controlled microporosity^31,32^. The type of activating agent notably affects the textural characteristics of the solid obtained. Optimal operating conditions vary significantly depending on the reactive agent used^33^.

Activation using KOH as the activating agent is an effective and popular method for the development of microporous carbonaceous materials with very high surface area and adsorption capacity, especially when they are derived from biomassic raw materials^34–40^. Since biopolymers constituting biomassic precursors are hydrolyzed in basic media, they are carbonized prior to impregnation with KOH solutions, to avoid disintegration and favour reaction with KOH^34–40^.

Textural characteristics of activated carbons are known to play a fundamental role in their adsorption performance, and therefore a comprehensive understanding and reliable characterization of their porous structures are relevant for practical applications. In a previous work Gomez Delgado et al.^41^ developed a series of activated carbons from Pine cones targeted at post-combustion CO_2_ capture through chemical activation with KOH, by varying the impregnation ratio and the final thermal treatment temperature. Textural characteristics of the developed activated carbons were assessed through the use of conventional methods applied to adsorption isotherms. Although the Brunauer–Emmett–Teller (BET)^42^ and Dubinin–Radushkevich (DR)^43^ models are commonly employed to determine the specific surface area and micropore volume, these methods present certain limitations, mainly because they assume that the adsorption process takes place on a homogeneous surface.

Within this framework, the aim of the present work is to deepen knowledge into the porous structures of the aforementioned Pine cones-derived activated carbons by applying the advanced new numerical clustering based adsorption analysis (LBET) method^44–48^, together with the Quenched Solid Density Functional Theory (QSDFT) method^49–55^. These methods, in contrast to the conventional ones, take into account the heterogeneity of the surface, among other factors, and are expected to lead to reliable textural parameters that contribute to an improved, more accurate representation of the developed porous structures.

## Materials and methods

The paper by Gomez-Delgado et al.^41^ presents the results of an analysis of the influence of preparation conditions, on the formation of the porous structure of activated carbons developed from Pine cones. The method used to prepare the activated carbons was described in detail in^41^. Briefly, the preparation process was carried out in two stages: the first consisted of the carbonization of the precursor material at 773 K under a N_2_ flow, while the second stage consisted of the thermal treatment of the carbonized precursor after being impregnated with KOH. In particular, the effects of the degree of impregnation, i.e., the ratio *R* of KOH to the carbonization product of the cones (*R* = 1, 2, and 3), and the activation temperature *T* (*T* = 873, 973, and 1073 K) on the development of the porous structure of the obtained activated carbons (ACL*R*, ACM*R* and ACH*R*, respectively), were analyzed^41^.

Porous structure analysis was performed based on nitrogen (N_2_) adsorption isotherms determined at 77 K, and carbon dioxide (CO_2_) adsorption isotherms determined at 273 K using Micromeritics ASAP 2020 HV for individual activated carbon samples. The morphological characterization of the precursor and the resulting activated carbons was performed by scanning electron microscopy (SEM), using a Zeiss Supra 40 electron cannon field emission microscope (Carl Zeiss AG, Oberkochen, Germany).

On the basis of adsorption isotherms determined, the values of specific surface area *S*_*BET*_ from the Brunauer–Emmett–Teller (BET) equation^42^, the total pore volume *V*_*t*_ from the volume of N_2_ adsorbed at maximum relative pressure *P/P*_*0*_ = 0.99, and the micropore volume *V*_*micro*_ from the Dubinin-Raduskevich (DR) equation were calculated^43^. Bearing in mind the well-known limitations of the BET and DR methods, especially the lack of consideration for the surface heterogeneity, a concept has been developed to extend the conducted studies with a comprehensive analysis of the porous structure based on nitrogen adsorption isotherms using the LBET^44–48^ and QSDFT^49–55^ methods.

The LBET method, the theoretical foundations of the LBET models and their derivations, as well as the numerical fast multivariate procedure for adsorption system identification are described in detail in earlier publications^44–48^. Therefore, only the most important information about the LBET method necessary for the correct interpretation of the results will be briefly presented, including a detailed discussion of the parameters of the implemented LBET models.

The LBET method considers the surface heterogeneity, the possibility of adsorbate molecule cluster branching, and the geometrical and energy limitations of the formation of clusters of adsorbate molecules. In this method, the process of adsorption on a heterogeneous surface is viewed as the clusterisation of adsorbate molecules in highly dispersed space limited by the geometry of micropores. The clusters are constructed by adding consecutive layers being in equilibrium with the volatile phase. The set of adsorbate molecules, which were adsorbed mainly due to adhesive interactions with the adsorbent matter, is treated as the first layer of adsorption. Joining of further adsorbate molecules is viewed as the second layer of adsorption^44–48^.

The adsorbate clusters considered in this theory are assumed to be constructed in configurationally independent ways, each starting with a unique primary site. In the LBET method, there exists a distinction between the two types of models^44–48^. The first model type refers to the adsorption system in which the limitation in the number of layers results from competing the physical adsorption. The second model type describes the systems in which limitations of cluster size result from pores size.

In addition, the LBET method enables the determination of the shape of the adsorption energy distribution on the surface of the material, thus giving much more information on the porous structure of activated carbons being analysed. In particular, the following porous structure parameters were determined using the LBET method and a method for fast multivariant identification of adsorption systems^44–48^: the dimensionless energy parameters *Q*_*A*_*/RT* and *B*_*C*_, corresponding to the maximum value of the adsorption energy on the first and subsequent layers, respectively; the volume of the first adsorbed layer *V*_*hA*_ (cm^3^/g) interpreted as the volume of space accessible for the first adsorption layer; the geometrical parameter of the porous structure *α* determining the height of the adsorbate molecule clusters, *α∈  *(0–1) and α = 0 meaning that in pores of the analysed material only individual molecules are present; the geometrical parameter of the porous structure *β* determining the width of the adsorbate molecule clusters; the surface heterogeneity parameter, *h*, expressing the energetical heterogeneity of the surface.

The energetical heterogeneity of the microporous adsorption systems significantly and negatively impacts the numerical conditioning of the system identification tasks. To solve this problem, a unique fast multivariate method for fitting the LBET class models to the adsorption isotherms was proposed, which is also employed to define the value of the surface heterogeneity indicator *h* and the shape of the distribution of adsorption energy on the first layer^44–48^.

Additionally, it is calculated the dispersion of fitting error, *σ*_*e*_, expressing the quality of fitting the theoretical adsorption isotherm to empirical data. To make possible application of the LBET method for near and supercritical temperatures, an original fluid state model was elaborated.

### Ethical approval

The pine cones samples were supplied by a logging company. They constitute a by-product of the activity. The forestry activity in Argentina is regulated by the Forest Law No. 26331, whose enforcement authorities are the Ministry of Agriculture, Livestock and Fisheries, and the Ministry of Environment and Sustainable Development of the Nation. The regulations of logging and forest care are included in the framework of the recommendations of International Union for Conservation of Nature (IUCN) Policy Statement on Research Involving Species at Risk of Extinction. Pinus canariensis has been categorized by IUCN, as a least-concern species. Besides, guidelines of the Convention on International Trade in Endangered Species of Wild Fauna and Flora (CITES) are carefully taken in account for the forest activity.

E. Gomez Delgado, G.V. Nunell, P.R. Bonelli, and A.L. Cukierman carried out the formal characterization of the Pine cones used in the present study, according to the keys to Pinus canariensis species as thoroughly outlined and illustrated in “A Handbook of the World’s Conifers” by Aljos Farjon (Volumes I and II, 2nd Revised edition, Brill, Leiden—Boston, pages 663–665, 2017).

No voucher specimen has been deposited in a publicly herbarium since there are only few pine species in South America, all being introduced, that can be easily identified.

## Discussion of the results

On the basis of previous results of nitrogen adsorption isotherms analyses carried out by means of BET and DR methods, it was shown that with the increase in both the degree of impregnation as well as the activation process temperature, both the specific surface area and the volume of micropores of the obtained activated carbons increased significantly (Table 1).

**Table 1:** Parameters characterizing the microporous structure of the activated carbons developed by chemical activation of Pine cones with KOH, obtained with different impregnation ratios and process temperatures, based on the analysis of N_2_ adsorption isotherms using the BET, DR, LBET, and DFT methods.

Sample	*S*_*BET*_ (m^2^/g)	*V*_*t*_ (cm^3^/g)	*V*_*micro*_ (cm^3^/g)	LBET M No.	*V*_*hA*_ (cm^3^/g)	*Q*_*A*_/*RT*	*B*_*C*_	*h*	*α*	*β*	*S*_*QSDFT*_ (m^2^/g)	*V*_*QSDFT*_ (cm^3^/g)
ACL1	395	0.18	0.18	30	0.156	− 12.90	1.00	9	0.00	1.00	482	0.157
ACL2	697	0.33	0.32	15	0.286	− 12.36	7.66	9	0.00	1.00	887	0.289
ACL3	1534	0.71	0.71	15	0.651	− 10.96	7.62	9	0.04	1.00	1823	0.655
ACM1	634	0.30	0.30	30	0.267	− 12.25	7.61	9	0.02	1.00	944	0.274
ACM2	996	0.47	0.47	15	0.498	− 12.13	7.66	9	0.03	1.00	1590	0.502
ACM3	1922	0.89	0.90	15	0.826	− 9.76	7.62	9	0.01	1.00	2210	0.832
ACH1	888	0.42	0.42	15	0.380	− 12.36	7.66	9	0.04	1.00	1214	0.384
ACH2	1060	0.52	0.52	15	0.464	− 11.30	7.59	9	0.02	1.00	1412	0.467
ACH3	2202	1.01	1.01	15	0.939	− 9.05	7.47	9	0.04	1.00	2285	0.937

Where: *S*_*BET*_—the specific surface area; *V*_*micro*_—the micropore volume; *V*_*t*_—the total pore volume; *V*_*hA*_—the volume of the first adsorbed layer, *Q*_*A*_/*RT*—the dimensionless energy parameter for the first adsorbed layer; *B*_*C*_—the dimensionless energy parameter for the higher adsorbed layers; *α*—the geometrical parameter of the porous structure determining the height of the adsorbate molecule clusters; *β*—the geometrical parameter of the porous structure determining the width of the adsorbate molecule clusters; *h*—the surface heterogeneity parameter; S_*QSDFT*_—the micropore specific surface area, *V*_*QSDFT*_—the volume of micropores.

Activated carbons obtained from Pine cones at impregnation ratio *R* = 3, were characterized by very large specific surface areas S_*BET*_ as well as micropore volumes *V*_*micro*_; the highest values of these parameters were determined for activated carbon ACH3 obtained at the highest activation temperature i.e. 1073 K (see Table 1).

The comparison of the micropore volume *V*_*micro*_ and total pore volume *V*_*t*_ also provides very interesting conclusions, which indicates that only micropores are present in the activated carbons analysed. However, the presence of micropores alone in the activated carbons analyzed, despite the fact that it is an extremely desirable feature in adsorption technologies, undermines the reliability of the results obtained using the BET method.

Application of the LBET method, also showed that increasing both the degree of impregnation *R* and the activation temperature, led to increase the values of *V*_*hA*_ parameters determined for individual activated carbon samples. On the other hand, the value of the energy parameter *Q*_*A*_*/RT* decreases significantly with increasing the value of the impregnation degree *R*.

The values of the heterogeneity parameter *h* determined for the analysed samples indicate that the surface of the obtained activated carbons from Pine cones is strongly heterogeneous. The fact that such a value was obtained for all the analysed activated carbon samples indicates that the morphological structure of the original raw material and of the char obtained from it had a significant impact on the porous structure. The values of geometrical parameters *α* and *β* indicate that only single nitrogen molecules adsorb in the micropores of the analysed material.

**Figure 1:**
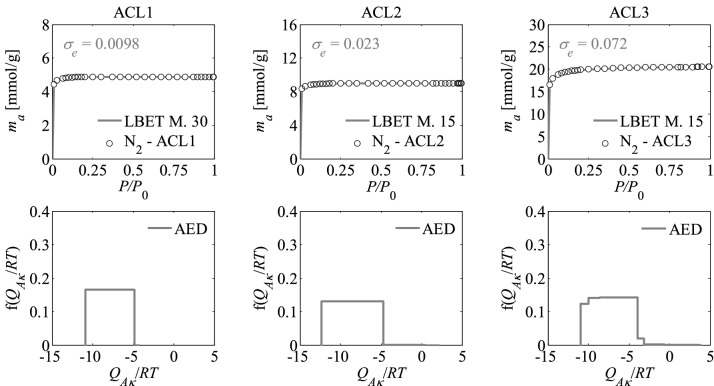
Nitrogen adsorption isotherms and adsorption energy distributions (AED) obtained by applying the LBET method to the experimental data for the activated carbons ACL*R* developed by chemical activation of Pine cones with KOH, at the lowest temperature i.e. *T* = 873 K with the different impregnations ratios (*R* = 1, 2, 3); *σ*_*e*_ is the dispersion of fitting error.

**Figure 2:**
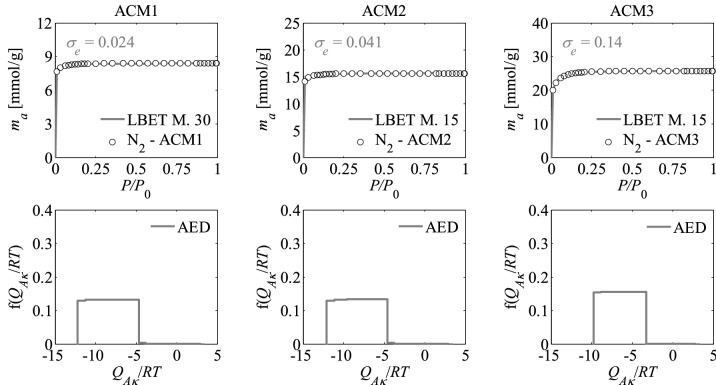
Nitrogen adsorption isotherms and adsorption energy distributions (AED) obtained by applying the LBET method to the experimental data for the activated carbons ACM*R* developed by chemical activation of Pine cones with KOH, at the middle temperature i.e. *T* = 973 K with the different impregnations ratios (*R* = 1, 2, 3).

**Figure 3:**
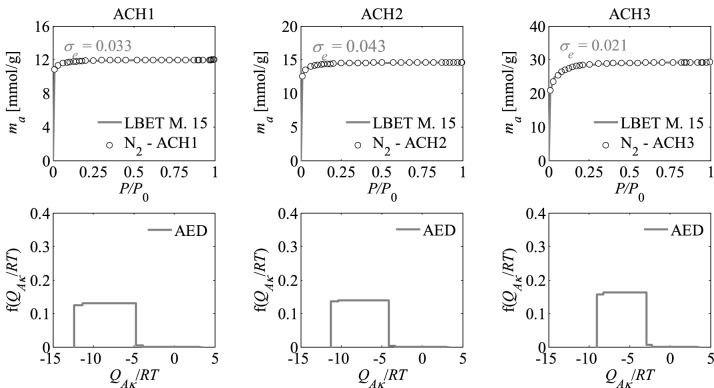
Nitrogen adsorption isotherms and adsorption energy distributions (AED) obtained by applying the LBET method to the experimental data for the activated carbons ACH*R* developed by chemical activation of Pine cones with KOH, at the highest temperature i.e. *T* = 1073 K with the different impregnations ratios (*R* = 1, 2, 3).

Nitrogen adsorption isotherms and adsorption energy distributions (AED) obtained by applying the LBET method to the experimental data for the activated carbons are presented in Figs. 1, 2 and 3.

The shapes of adsorption energy distribution (AED) for the first adsorbed layer determined for the analysed activated carbon samples based on the nitrogen adsorption isotherms indicate the occurrence of a wide range of adsorption energy on the first adsorbed layer.

**Figure 4:**
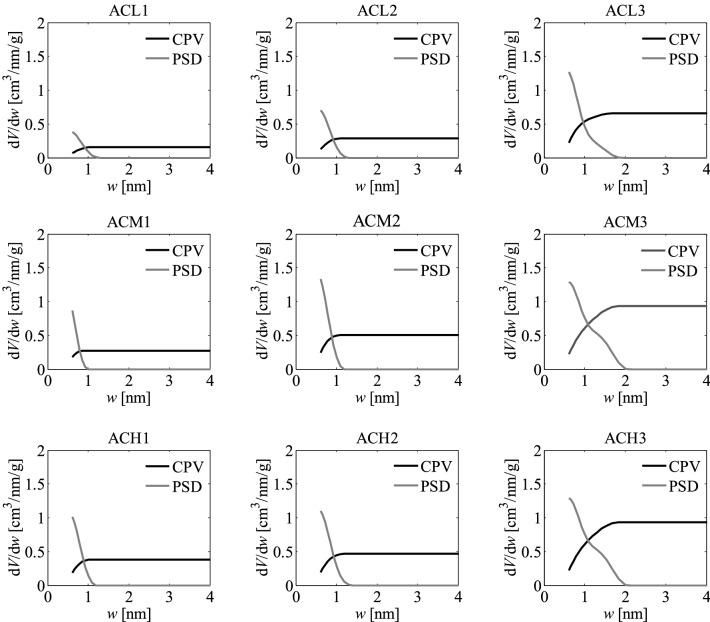
Pore size distributions (PSD) and cumulative pore volumes (CPV) obtained for the activated carbons developed by chemical activation of Pine cones with KOH, obtained with the different temperatures and impregnation ratios, by applying the QSDFT method to the corresponding nitrogen adsorption isotherms.

As part of the research, pore size distributions (PSD) and cumulative pore volumes (CPV) were also determined based on the nitrogen adsorption isotherms using the QSDFT method^49–55^. They are presented in Fig. 4. The results show that both experimental variables investigated, namely the activation temperature and impregnation ratio, had influence on the development of pores and their characteristics. At constant temperature, increasing the impregnation ratio led to a greater amount of pores in the matrix of the activated carbons, as shown in the corresponding cumulative pore volume plots.

The PSDs of the activated carbons obtained with *R* = 1 or *R* = 2 reached diameters between 1.0 and 1.4 nm, but when *R* rose to 3 the upper limit of the PSD extended to ~ 2.0 nm. This can be attributed to the widening of the existing pores likely due to the rupture and weakening of the walls of the porous matrix, as might be inferred from certain signs of fragmentation and disintegrated particles in the SEM images for the activated carbons obtained with the highest *R*, that are later shown in Fig. 9. Accordingly, it appears that, at constant temperature, there was an over-activation effect when *R* = 3 was used, resulting in a widening of the distributions towards larger pore sizes.

Likewise, as can be inferred from the cumulative pore volume plots, at constant *R*, the presence of micropores rises with increase in the temperature, even though the upper limits of the PSDs remain unchanged, thus indicating that the PSD broadness is more sensitive to changes in *R*.

The PSDs plots indicate that all the samples are microporous, showing pores especially in the supermicroporous range (0.7 nm–2 nm). This result is consistent with the pore diameter calculated using the N_2_ isotherm since for all samples this diameter is around 1.8 nm, being the smallest diameter that can be obtained from the information given by the N_2_ isotherm. This would also be in agreement with the data presented in Table 1, showing that the total volume of pores (*V*_*t*_) and the volume of micropores (*V*_*micro*_) are similar for all the activated carbons.

**Figure 5:**
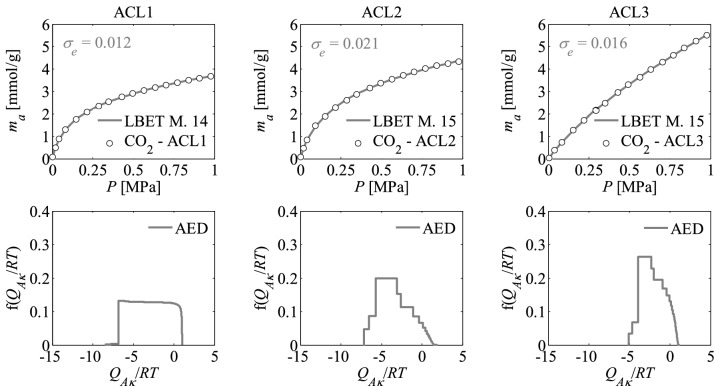
Carbon dioxide adsorption isotherms and adsorption energy distributions (AED) obtained by applying the LBET method to the experimental data for the activated carbons developed by chemical activation of Pine cones with KOH, at the lowest temperature (ACL*R*) with the different impregnations ratios (*R* = 1, 2, 3).

**Figure 6:**
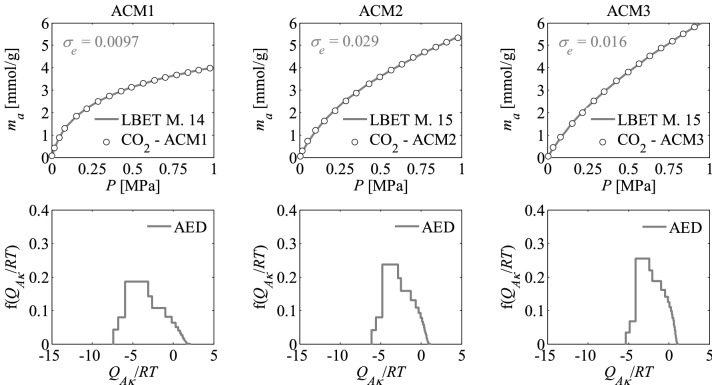
Carbon dioxide adsorption isotherms and adsorption energy distributions (AED) obtained by applying the LBET method to the experimental data for the activated carbons developed by chemical activation of Pine cones with KOH, at the middle temperature (ACM*R*) with the different impregnations ratios (*R* = 1, 2, 3).

**Figure 7:**
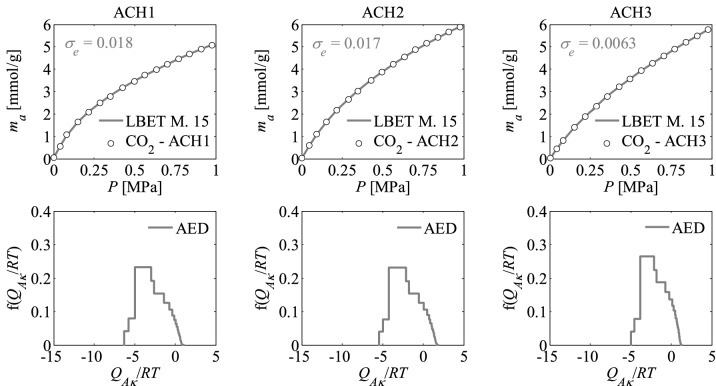
Carbon dioxide adsorption isotherms and adsorption energy distributions (AED) obtained by applying the LBET method to the experimental data for the activated carbons developed by chemical activation of Pine cones with KOH, at the highest temperature (ACH*R*) with the different impregnations ratios (*R* = 1, 2, 3).

**Figure 8:**
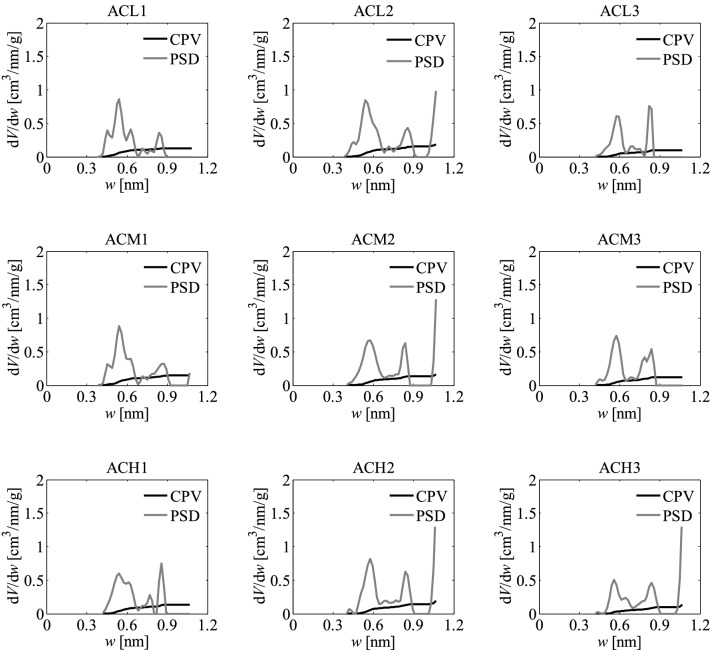
Pore size distributions PSD and cumulative pore volumes CPV obtained for the activated carbons developed with the different temperatures and impregnation ratios, by applying the QSDFT method to the corresponding carbon dioxide adsorption isotherms.

Physical adsorption of N_2_ at 77 K is widely used for porosity characterization, because it covers a wide relative pressure range (from 10^–8^ to 1 *P*/*P*_*0*_). However, it has the disadvantage of possible problems of N_2_ diffusion inside the narrow porosity (size < 0.7 nm). To overcome this problem, the use of other adsorbates at higher temperatures has been proposed. Carbon dioxide CO_2_ adsorption at 273 K is considered an easy alternative to N_2_ for the assessment of such narrow microporosity^56^. Despite the larger dimension of CO_2_ molecules compared to N_2_, the higher adsorption temperature, in contrast to N_2_ at 77 K, results in a larger kinetic energy of the CO_2_ molecules, that are able to enter into the narrow pores, thus avoiding the diffusional problems above mentioned. Therefore, in the present study, carbon dioxide adsorption isotherms determined at 273 K were additionally analyzed for all the obtained activated carbon samples from Pine cones. The DR, LBET, and QSDFT methods were used for the calculations analogously to the nitrogen adsorption isotherms, and the results are summarized in Table 2 and shown in Figs. 5, 6, 7, 8.

**Table 2:** Parameters characterizing the microporous structure of the activated carbons developed by chemical activation of Pine cones with KOH, obtained with different impregnation ratios and process temperatures, based on the analysis of CO_2_ adsorption isotherms using the DR, LBET, and QSDFT methods.

Sample	*V*_*micro*_ (cm^3^/g)	LBET M No.	*V*_*hA*_ (cm^3^/g)	*Q*_*A*_/*RT*	*B*_*C*_	*h*	*α*	*β*
ACL1	0.28	14	0.351	− 8.41	1.00	9	1.00	1.77
ACL2	0.34	15	0.396	− 7.13	2.32	9	0.81	4.00
ACL3	0.52	15	0.807	− 5.11	1.10	9	0.90	3.97
ACM1	0.30	15	0.365	− 7.37	3.06	9	0.81	4.08
ACM2	0.47	15	0.552	− 6.09	1.11	9	0.86	4.00
ACM3	0.56	15	0.845	− 5.34	1.10	9	0.90	3.51
ACH1	0.42	15	0.482	− 6.31	1.06	9	0.86	4.10
ACH2	0.54	15	0.899	− 5.43	2.36	9	0.87	4.00
ACH3	0.53	15	0.952	− 5.00	1.36	9	0.88	2.83

As can be seen from the results collected in Table 2, as both the activation temperature and the degree of impregnation increase, the value of the parameter *V*_*micro*_*, i.e.* the volume of micropores calculated from the DR equation, increases; similarly, the values of the parameter *V*_*hA*_, i.e. the volume of the first absorbed layer calculated using the LBET method, also increases. It is pointed out that the increase in the values of the mentioned parameters is more influenced by the degree of impregnation compared to the influence of the temperature of the activation process.

The values of the energy parameters for the first layer as well as for the higher layers, i.e. *Q*_*A*_/*RT* and *B*_*C*_, respectively, indicate strong interactions of the first layer with the surface of the tested activated carbon samples and much smaller interactions between the subsequent adsorbed layers.

In turn, the values of the surface heterogeneity parameter *h* determined for individual activated carbon samples indicate that the surface of these materials is strongly energetically heterogeneous. However, the values of geometric parameters α and *β* for most samples are practically similar to each other and indicate that high and significantly branching clusters of carbon dioxide molecules are formed in the micropores of these materials.

The exceptions are samples ACL1 and ACH3 for which lower values of geometric parameters *β* were obtained. This is related to the fact that in the case of ACL1, the microporous structure did not develop significantly, which was influenced by both the low degree of impregnation and the relatively low temperature of the activation process. On the other hand, in the case of activated carbon marked as ACH3, a lower value of the parameter *β,* in comparison with the other samples indicates the formation of narrower clusters of carbon dioxide molecules, while the structure is more developed, which is shown by the highest value of the *V*_*hA*_ parameter among all the samples analyzed.

Comparing the results obtained from the analysis of carbon dioxide adsorption isotherms with those obtained from nitrogen adsorption isotherms, an earlier suspicion that the porous structure of the studied activated carbons samples may contain pores inaccessible to nitrogen molecules, but accessible to carbon dioxide molecules, was confirmed.

The values of energy parameters and especially geometric parameters of LBET method determined for carbon dioxide adsorption isotherms are very similar, so it can be concluded that in the case of the primary raw material used, its morphological structure has a significant effect on the development of the porous structure of the obtained activated carbons.

The pore size distributions PSD and cumulative pore volumes CPV, shown in Fig. 8, were also determined as part of the work conducted.

Based on these pore size distributions, it can be seen that the range from 0.3 to 0.9 nm is found for all the analyzed activated carbons, that have a bimodal structure of the smallest pores. This observation indicates the significant influence of the morphological structure of the original raw material on the formation of the microporous structure of the obtained activated carbons, as stated earlier. The mentioned feature of Pine cones as a primary material opens up many possibilities for obtaining adsorbents with unique porous structures, including those designed for carbon dioxide sequestration processes.

**Figure 9:**
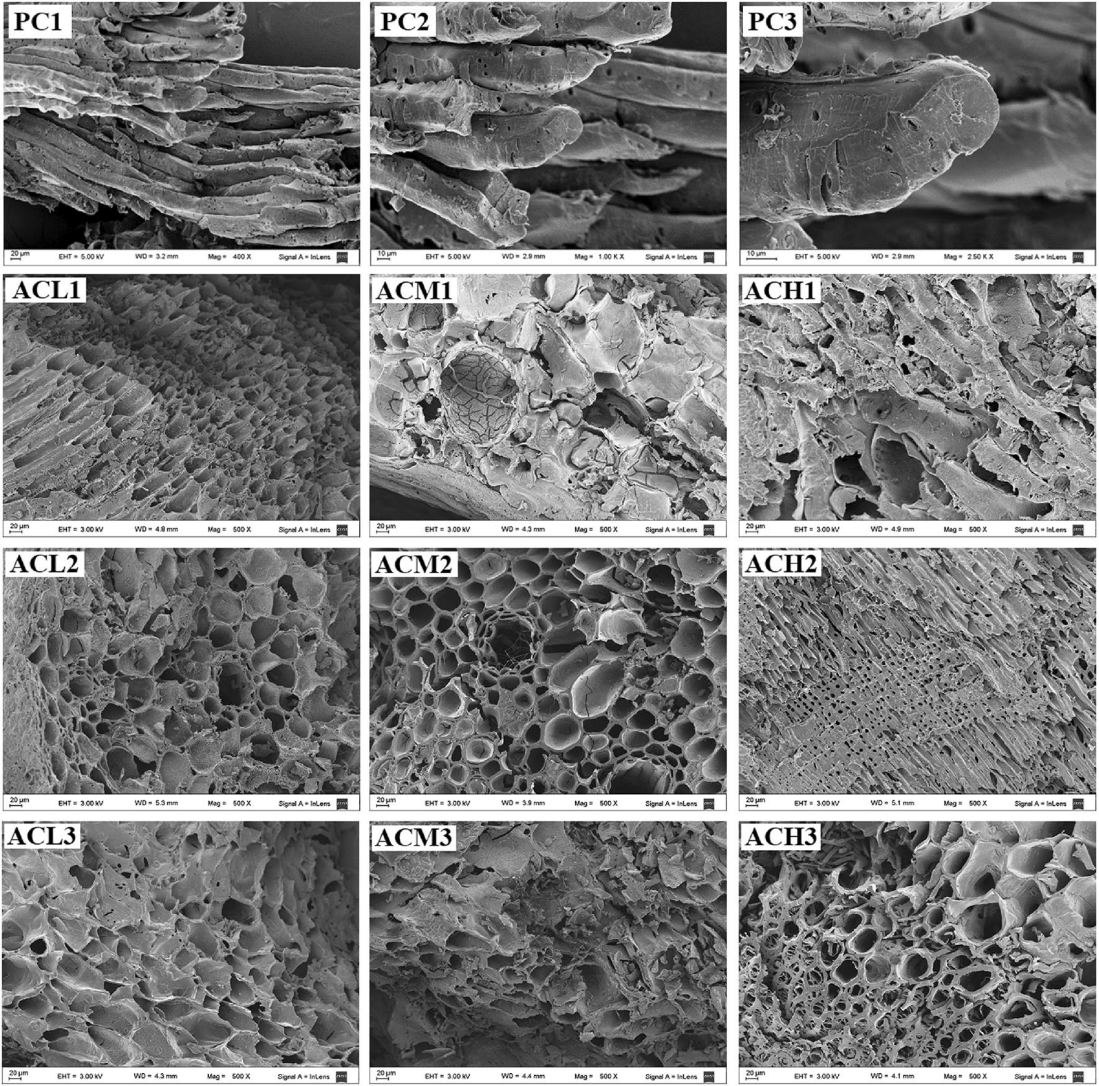
SEM images of Pine cones (PC) at different magnifications: PC1 (× 500), PC2 (× 1000) and PC3 (× 2500), and of the activated carbons (× 500) developed with different impregnation ratios and activation temperatures.

Figure 9 displays SEM images obtained for the Pine cones and the developed activated carbons. Micrographs of the precursor reveal typical morphological features of lignocellulosic materials, commonly observed in softwoods^57^. The presence of simple and fairly uniform structures, composed of few types of elongated fibrous cells, i.e. tracheids, may be noticed. Comparison of the SEM images of the developed ACs with those of the precursor, indicates that the activation process promoted an increase in the cavities on the outer surface of the cell wall in all cases. The activated carbons show spongiform structures that present certain similarity to those of a honeycomb with some openings and rough terminations.

The presence of particles of different sizes and shapes can also be observed, possibly originated from fragmentation of the cell wall. On the other hand, it may be noticed that part of the original morphology of the precursor remained preserved after the activation process. This fact is more evident for the sample obtained with the lowest temperature and impregnation ratio (ACL1). In this image, it is even possible to visualize a fibrous structure composed of long channels arranged in parallel, showing that the directional pattern of the precursor vessels remained practically intact and without signs of structural collapse. Overall, the SEM images indicate that more severe conditions led to intensify the development of the porous structures of the activated carbons.

Furthermore, the effect of the chemical nature of the activator on the formation of the porous structure of activated carbons was also analyzed. For this purpose, the results of calculations for the activated carbons obtained by KOH activation were compared with the results of analyses for activated carbons obtained from the same precursor by H_3_PO_4_ activation by Nunell et al.^58^. In the mentioned work, the activated carbons were obtained by activation of Pine cones using a 50% solution of H_3_PO_4_ acid, at a weight ratio of activator to precursor equal to 2, at 723 K (carbons designated as ACP).

The resulting activated carbons were also subjected to additional heat treatment at 1073 K in an inert N_2_ atmosphere (activated carbons designated as ACPT) and to impregnation with urea aqueous solution and subsequent heat treatment at 623 K (activated carbons designated as ACPU)^58^. The nitrogen adsorption isotherms were determined for the activated carbons obtained in this way and their structures were analyzed using BET, LBET and QSDFT methods. The results are summarized in Table 3.

**Table 3:** Parameters characterizing the microporous structure of the activated carbons obtained by phosphoric acid activation (ACP) and subjected to thermal (ACPT) and chemical (ACPU) post-treatments, based on the analysis of N_2_ adsorption isotherms using the BET, LBET, and QSDFT methods.

Sample	*S*_*BET*_ (m^2^/g)	*V*_*t*_ (cm^3^/g)	LBET M No.	*V*_*hA*_ (cm^3^/g)	*Q*_*A*_/*RT*	*B*_*C*_	*h*	*α*	*β*
ACP	1082	0.7	13	0.699	− 10.15	4.79	9	1.00	1.00
ACPT	772	0.4	23	0.430	− 13.54	5.94	5	0.99	1.00
ACPU	449	0.2	17	0.202	− 11.16	5.25	1	1.00	1.00

From the results obtained, it can be concluded that the activated carbon designated as ACP has a relatively large *S*_*BET*_ specific surface area and a large pore volume. It was also shown from the LBET analysis that the volume of the first adsorbed layer defined by the parameter *V*_*hA*_ is large. The values of energy parameters *Q*_*A*_*/RT* and *B*_*C*_ point to favorable conditions for the occurrence of the multilayer adsorption process, and the value of parameter *h* indicates that the surface of the activated carbon obtained with the acid is strongly heterogeneous, showing the same value than the KOH-derived carbons.

The values of *h* decreases markedly for the post-treated activated carbons, thus indicating that the additional treatments promote strong modifications in the surface heterogeneity of the phosphoric acid-derived activated carbon. On the other hand, the values of geometrical parameters *α* and *β* indicate that high and non-branching clusters of adsorbate molecules are formed in the pores of this adsorbent.

ACPT activated carbon subjected to an additional heat treatment is characterized by significantly lower values of *S*_*BET*_, *V*_*t*_ and *V*_*hA*_ parameters while having a lower degree of surface heterogeneity compared to the ACP sample without additional heat treatment (see Table 3). The ACPU activated carbon obtained by impregnation of ACP with an aqueous solution of urea and successive heat treatment is characterized by significantly lower values of *S*_*BET*_, *V*_*t*_ and *V*_*hA*_ parameters while having the lowest degree of surface heterogeneity among the analyzed samples.

**Figure 10:**
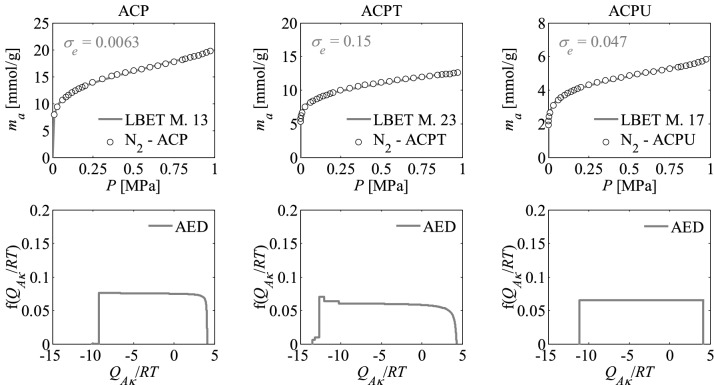
Nitrogen adsorption isotherms and adsorption energy distributions (AED) obtained by applying the LBET method to the experimental data for the activated carbons developed by the process of activation with phosphoric acid (ACP) and subjected to an additional method of thermal (ACPT) and chemical (ACPU) treatments.

**Figure 11:**
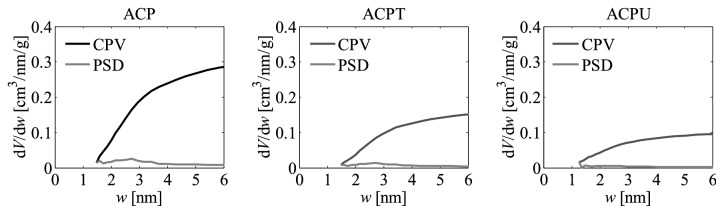
Pore size distributions (PSD) and cumulative pore volumes (CPV) obtained for the activated carbons developed with phosphoric acid activation and subjected to additional thermal (ACPT) and chemical (ACPU) treatments, by applying the QSDFT method to the corresponding nitrogen adsorption isotherms.

Figure 10 shows nitrogen adsorption isotherms and adsorption energy distributions (AED) obtained by applying the LBET method to the experimental data for the activated carbons prepared by chemical activation with phosphoric acid (ACP) at pre-established experimental conditions, and for the samples arising from the thermal (ACPT) and chemical (ACPU) post-treatments. Besides, pore size distributions and cumulative pore volumes calculated for these samples are displayed in Fig. 11.

The results clearly attest the pronounced influence of the activator used on the porous structure, indicating that phosphoric acid activation of the Pine cones led to development of larger pore sizes, mostly within the mesopore range, that remained in the post-treated samples although with a reduction in the pore volumes.

## Comparison of research results with those obtained in previous publications

In this section the effect of the biomass primary raw material on the formation of the porous structure of activated carbons in the process of activation with potassium hydroxide is analyzed. For this purpose, the results presented above were compared with those obtained for activated carbons made from a variety of biomass raw materials by activation with KOH, reported in earlier publications^59–61^.

The results in^59^ include an analysis of the porous structure of activated carbons made from demineralized Kraft lignin by chemical activation using, inter alia, potassium hydroxide KOH. The activation was carried out at different temperatures *T*, i.e. 873 K, 973 K and 1073 K, with a mass ratio *R* equal to 3, and with different mass ratios of *R* hydroxide to raw material equal to 1, 2 and 3, at an activation temperature *T* = 973 K^59^.

Comparing the results of the porous structure analysis for the activated carbons obtained from Kraft lignin and Pine cones, a significant influence of the primary raw material on the formation of the structure of activated carbons and their adsorption properties may be inferred. Namely, comparing in particular the parameters of the porous structure of activated carbon obtained from Kraft lignin at 873 K, with a mass ratio of *R* = 3, it can be noted that the said activated carbon is characterized by a significantly higher specific surface area determined from the BET equation and the volume of micropores. Likewise, the comparison indicates a higher adsorption energy for the first adsorbed layer as well as a significantly higher value of the volume of the first adsorbed layer and a significantly lower degree of surface heterogeneity with respect to the activated carbon obtained from Pine cones under identical preparation conditions.

There are also significant differences in the determined values of the geometric parameters of the LBET models. Namely, the values of the mentioned parameters calculated for the activated carbon obtained from Kraft lignin by KOH activation at 873 K, with a mass ratio of 3, indicate that very high and unbranched clusters of nitrogen molecules are formed in the micropores of this material. In contrast, the values of geometric parameters determined for activated carbon obtained from Pine cones under the same preparation conditions indicate that only single adsorbate molecules are adsorbed in the pores. In the case of activated carbons made from Kraft lignin, at the activation process temperature of *T* = 973 K, with a mass ratio of *R* = 3, a very high value of specific surface area, volume of micropores, as well as volume of the first adsorbed layer was obtained, not only in comparison with the sample of activated carbon obtained from Pine cones under the same conditions but also in comparison with activated carbon obtained from Kraft lignin at 1073 K^59^.

Analysis of the porous structure of activated carbons obtained at 1073 K also leads to interesting conclusions. As already mentioned in the case of activated carbons obtained from Kraft lignin, the sample obtained at this temperature was characterized by lower values of specific surface area, volume of micropores and volume of the first adsorbed layer, indicating the destructive effect of higher temperature on the pore structure, i.e. on the burning of walls between adjacent pores. The situation is different for activated carbons obtained from Pine cones, namely, at 1073 K, activated carbons with the largest values of specific surface area, volume of micropores and volume of the first adsorbed layer were obtained among carbons obtained from Pine cones. It is also worth noting that samples of activated carbons obtained from Kraft lignin were characterized by significantly lower degrees of surface heterogeneity compared to samples obtained from Pine cones, which, regardless of the temperature of the activation process, had the highest degree of surface heterogeneity (*h* = 9).

Analysis of adsorption energy distributions determined by the LBET method and pore size distributions determined by the QSDFT method also provide important information. As shown in the article^59^, the shapes of adsorption energy distributions on the first layer determined for activated carbons obtained from Kraft lignin indicate the existence of a wide spectrum of medium-energy sites. In contrast, activated carbons obtained from Pine cones showed the existence of a significant proportion of high-energy sites.

Equally important information about the analysed materials was provided by pore size distributions determined by the QSDFT method, namely, it was shown that activated carbons obtained from Kraft lignin were characterized by a wide pore size distribution covering a range up to about 4 nm, while activated carbons obtained from Pine cones, covered a range limited only to micropores, i.e. up to 2 nm.

Interesting results are also provided by a comparison of the obtained structure parameters for activated carbons obtained from Kraft lignin and Pine cones at the activation process temperature *T* = 973 K and at mass ratios of activator to activated substance *R* = 2 and 3. As shown, the activated carbon prepared from Kraft lignin at 973 K and at a mass ratio *R* equal to 2 was characterized by a very high value of the specific surface area parameter—more than two and a half times that of the activated carbon prepared from Pine cones under the same preparation conditions. Activated carbon obtained from Kraft lignin, moreover, was characterized by twice the pore volume compared to activated carbon obtained from Pine cones at virtually identical volume of the first adsorbed layer. However, it is noteworthy that adsorbent obtained at 973 K from Kraft lignin at a mass ratio *R* equal to 2 was characterized by a very high degree of surface heterogeneity (*h* = 9), identical to those obtained from Pine cones. However, activated carbon obtained from Kraft lignin at the same temperature and mass ratio *R* = 3, was characterized by a much lower degree of surface heterogeneity.

Significant differences were also observed in the shapes of the adsorption energy distribution on the first layer for activated carbons prepared from Kraft lignin obtained at different mass ratios, in contrast to activated carbons obtained from Pine cones, for which the energy adsorption distributions (AED) are characterized by virtually identical shapes. A comparison of the pore size distributions (PSD) determined for the analysed activated carbons also provided interesting observations, namely, activated carbons obtained at *R* = 2 were characterized by a much narrower range of pore sizes, compared to activated carbons prepared at a mass ratio of *R* = 3.

Another article^60^ presents the results of analysis of the structure of activated carbons produced from Pistachio nut, Hazelnuts and Pecan nuts shells. These adsorbents were obtained by a two-step process, i.e. carbonization at 773 K and KOH activation with mass ratios *R* = 1, 2, 3 and 4 at an activation process temperature of 1073 K. Comparing the results of the analysis of the structure of activated carbons prepared from the shells of the three kind of nuts with the results obtained for Pine cones activated carbons, it is noteworthy that the values of specific surface area and volume of the first adsorbed layer are significantly smaller, as well as the greater heights of clusters of adsorbate molecules in the case of activated carbons made from Pine cones. It is noteworthy that activated carbon with the largest volume of micropores, however, was obtained from Pine cones at a mass ratio of *R* = 3.

Activated carbons obtained from the shells of the three kind of nuts were characterized by a high or very high degree of surface heterogeneity^60^ but lower than for the activated carbons produced from Pine cones under the same preparation conditions. However, the exception was the activated carbon obtained from pecan shells at a mass ratio *R* of 3 and at an activation process temperature of 1073 K. This sample was characterized by the lowest degree of surface heterogeneity^60^.

The analysis of the shapes of adsorption energy distributions (AED) on the surface of the tested adsorbents and the determined pore size distributions also revealed differences in the porous structure of the obtained activated carbons related to the different structure of the primary raw material. Namely, the adsorption energy distributions determined for activated carbons obtained from Nut shells showed a predominant share of sites with high adsorption energy, while the aforementioned distributions determined for carbons obtained from Pine cones were characterized by a homogeneous distribution of sites with different energies. Some analogies between the porous structure of activated carbons obtained from Walnut shells and Pine cones were observed for the shape of the pore size distributions PSD. Namely, for lower mass ratios, much narrower PSDs were determined; however, the distributions determined for activated carbons prepared from Nut shells were bimodal, i.e., there were two peaks on the distributions corresponding to the proportions of dominant micropores and with an increase in the mass ratio of the activator to the activated substance, the proportion of larger micropores and mesopores increased, in contrast to activated carbons obtained from Pine cones, in which only micropores are present. However, if one compares activated carbons obtained from Nut shells at *R* = 1 and 2 with adsorbents obtained from Pine cones at mass ratios of 1, 2, and 3 respectively, one can see similarities in the pore size distribution. These similarities indicate not only similarities in the structure of the biomass material, but also the significant influence of the activator on the formation of the porous structure.

The article by Kwiatkowski and Broniek^61^ presents the results of a study of the structure of activated carbons prepared from Mahogany, Ebony and Hornbeam wood, through a carbonization process at 773 K and a chemical activation process with potassium hydroxide at 1073 K, with mass ratios *R* = 1, 2 and 3. The aforementioned activated carbons obtained from high-density biomass materials were characterized by very high values of specific surface area, volume of micropores, and volume of the first adsorbed layer, which is not surprising given the properties of the primary raw material^61^. However, an interesting observation made in the aforementioned article was that as the mass ratio *R* increased from 1 to 2, the degree of surface heterogeneity increased, while already at a mass ratio *R* equal to 3, the surface heterogeneity decreased significantly. In comparison, in the case of activated carbons obtained from Pine cones, the degree of heterogeneity was constant and independent of the preparation conditions, which clearly indicates the specific properties of the original raw material.

Another feature of activated carbons resulting from the characteristics of the primary raw material is the shape and size of the clusters of adsorbate molecules are formed. In the case of activated carbons obtained from hard wood species at *R* = 1, medium-height clusters of adsorbate molecules were formed, and at a mass ratio of *R* = 2, significantly higher clusters of nitrogen molecules were already formed, while already at *R* = 3 in the case of activated carbons obtained from Ebony wood and Hornbeam wood, only single nitrogen molecules were adsorbed in the pores^61^. There are also significant differences in the shapes of adsorption energy distributions (AED) determined for activated carbons obtained from different woods and Pine cones, namely, in the case of activated carbons obtained from Mahogany, Ebony and Hornbeam woods, with a mass ratio of *R* = 1, the shapes of the AED plot indicate the dominant contribution of high-energy sites. On the other hand, for activated carbons obtained from Mahogany and Ebony wood at a mass ratio of *R* = 2, the shape of the adsorption energy distribution shows a dominant share of medium adsorption energy sites. While the shapes of the AEDs plots for activated carbons obtained from different types of wood at *R* = 3 indicate the presence of a wide range of adsorption sites with different adsorption energies^61^. In contrast, in the case of activated carbons obtained from Pine cone, the shapes of the AEDs plots point to the presence of a medium-width spectrum of sites with different adsorption energies on the first layer. Note that the shapes of the adsorption energy distributions for activated carbons obtained from Pine cones at different *R* mass ratios are very close to each other, indicating the dominant influence of the primary raw material on the formation of their adsorption properties.

A comparative analysis of the shapes of the PSDs plots determined for activated carbons obtained from different types of wood and Pine cones indicates both some analogies and differences due to the different structure of the primary raw material from which the said carbons were obtained. In the compared activated carbons, as the mass ratio of activator to activated substance increased, an increase in distribution width was observed, covering an increasingly wide range of pore sizes. However, the distributions determined for activated carbons obtained from Mahogany, Ebony and Hornbeam wood, have a bimodal structure of micropores, and with an increase in the mass ratio *R*, there is a successive increase in the proportion of larger micropores and small mesopores, i.e., up to about 4 nm^61^. In the case of activated carbons obtained from Pine cones, analogously, as the mass ratio value increased, the width of the PSD plot increased, covering, however, at *R* = 3 the maximum only the range of micropores, i.e., up to 2 nm.

Based on the analysis of the various experimental results presented above, it is shown that using identical preparation conditions, activated carbons with different adsorption properties are obtained from different biomass materials, which indicates that the nature of the primary raw material has a dominant influence on the formation of the porous structure of the activated carbons obtained from it. Therefore, it is reasonable to search for new materials for the production of activated carbons, especially of biomass origin, which is the subject of many research works. However, it should be emphasized that in order to properly select preparation conditions taking into account the nature of the primary raw material to obtain a carbon adsorbent with specific adsorption properties, a detailed study of the effect of preparation methods and conditions on the formation of the porous structure is required. Such studies require suitably precise and reliable methods for assessing adsorption properties and the forming porous structure, especially the complementary analysis by LBET and QSDFT methods.

As part of the analysis, the unique characteristics of activated carbons obtained from Pine cones were demonstrated, including the presence of only micropores in their structure, thanks to which they can be used in advanced adsorption techniques and technologies, including in particular carbon dioxide sequestration.

## Conclusion

A thorough analysis of the porous structures of activated carbons developed by chemical activation of Pine cones with KOH has been carried out by applying novel advanced numerical methods to N_2_ and CO_2_ adsorption isotherms obtained for their characterization. The LBET analysis applied showed that the activated carbons were characterized by a very high degree of energy heterogeneity, very large values of the volume of the first adsorbed layer as well as specific surface areas. The analysis of nitrogen adsorption isotherms showed that single adsorbate molecules adsorb in the pores of the studied activated carbons. They also exhibited huge amounts of narrow micropores, as characterized in depth by the analysis applied to CO_2_ adsorption isotherms. This feature is of significant importance for the potential use of the analysed samples in carbon dioxide sequestration and other processes.

The analyses also showed a significant advantage of the LBET method over popular methods of porous structure analysis based on BET, and DR methods. The LBET method made it possible to determine the size of micropores and the degree of surface heterogeneity. Nevertheless, it should be kept in mind that it is only an approximation of the real porous structure based to a large extent on the adsorption of its specific mathematical model as well as an approximate model of the mechanism of adsorption processes occurring on the surface of adsorbents. There are very complicated mechanisms occurring during the processes of chemical activation, and thus practically no possibility to predict them. Therefore, in order to obtain adsorbents with appropriate parameters of the porous structure, it is necessary to perform a series of experiments supplemented by a reliable analysis of the structure, using advanced methods.

## Data Availability

All data generated or analysed during this study are included in this published article.
